# First report of polymorphisms and genetic characteristics of the *prion-like* protein gene (*PRND*) in bats

**DOI:** 10.3389/fvets.2026.1967863

**Published:** 2026-10-19

**Authors:** Da-In Choi, Min-Ju Jeong, Eun-Jee Na, Jae-Ku Oem, Byung-Hoon Jeong

**Affiliations:** 1 Korea Zoonosis Research Institute, Jeonbuk National University, Iksan, Republic of Korea; 2 Department of Bioactive Material Sciences and Institute for Molecular Biology and Genetics, Jeonbuk National University, Jeonju, Republic of Korea; 3 Laboratory of Veterinary Infectious Diseases, College of Veterinary Medicine, Jeonbuk National University, Iksan, Republic of Korea

**Keywords:** bats, Doppel protein, polymorphism, prion, *prion-like* protein gene, *PRND*, SNP

## Abstract

**Background:**

Prion diseases are fatal brain disorders caused by the misfolding of the normal cellular prion protein (PrP^C^) into its infectious isoform (PrP^Sc^). Previous studies have reported that many polymorphisms within the *prion-like* protein gene (*PRND*), a prion protein family member, are significantly associated with disease susceptibility in various species. There have been no natural cases of prion disease found in bats. The genetic polymorphisms of the *PRND* gene in bats commonly found in Korea were characterized for the first time in this study.

**Methods:**

A total of 98 bat samples were used to extract genomic DNA to investigate bat *PRND* polymorphisms. Next, amplicon sequencing focusing on the coding region of the bat *PRND* gene was conducted. To assess the linkage among the *PRND* single nucleotide polymorphisms (SNPs), linkage disequilibrium (LD) analysis was performed. The LD analysis was also conducted to investigate genetic associations between *PRND* and prion protein gene (*PRNP*) SNPs. In addition, we conducted sequence alignment and evolutionary analysis with ClustalW and MEGA 12.1, and assessed the effects of non-synonymous SNPs on Doppel protein using *in silico* tools.

**Results:**

We found 7 novel SNPs in the bat *PRND* gene. No pairwise LD value between the analyzed *PRNP* and *PRND* polymorphisms exceeded the threshold for strong LD (*r*^2^ > 0.333). Furthermore, according to preliminary computational analyses (SIFT and MutPred2), one non-synonymous SNP, c.392G>C (R131T), was predicted to have a potentially deleterious effect on bat Doppel protein.

## 1 Introduction

Prion diseases, characterized by the aggregation of pathological prion protein (PrP^Sc^) and vacuolation in the brain, can originate sporadically, by transmission, or by inheritance (1). Creutzfeldt-Jakob disease (CJD) in humans, and scrapie, bovine spongiform encephalopathy (BSE), chronic wasting disease (CWD) in animals are known as prion diseases (2, 3). At present, there are no effective treatments for prion diseases, which invariably lead to death.

*Prion-like* protein gene (*PRND*) is a key member of the mammalian prion gene family (4, 5). *PRND* is especially significant due to its close genomic proximity to the prion protein gene (*PRNP*), as well as the physiological and structural similarities between its product, the *prion-like* protein (Doppel), and the prion protein (PrP) (4). Doppel protein, as with PrP, connects to the outside of cell surfaces by a glycosylphosphatidylinositol (GPI) anchor (6). A previous study suggests that *PRND* controls signaling networks and gene expression events that collectively contribute to blood-brain barrier formation (7). Doppel protein has been implicated in neurotoxicity and may interact functionally with PrP, suggesting a potential role in prion pathogenesis. Polymorphisms in prion disease-associated genes also provide valuable information for understanding the mechanism of prion diseases. Previous studies have shown *PRND* polymorphisms in humans, cattle, sheep, goats, cats, and horses (8–12). Notably, polymorphism distribution at the +28 site within the 3′ untranslated region of the human *PRND* gene significantly differed between Korean sporadic CJD patients and healthy controls (13). There are no studies on *PRND* polymorphism in bats.

Bats are increasingly recognized as hosts of zoonotic and major zoonotic pathogens (14). In several countries, there exists a cultural practice of consuming bats as food or medicine (15, 16). BSE, an epizootic prion disease originating in cattle, was responsible for variant CJD in humans due to dietary exposure (17). This phenomenon underscores that animal consumption can facilitate pathogen transmission (18). As of now, there is no evidence of prion infection in bats. However, it is unknown whether bats are prion disease-susceptible or resistant species.

In the present study, we investigated the bat *PRND* genotype, allele, and haplotype frequencies of 7 single nucleotide polymorphisms (SNPs). Our research also involved analyzing linkage disequilibrium (LD) between polymorphisms of *PRNP* and *PRND*, and measuring the physical map distance separating these two genes. Furthermore, we predicted the 3D structures of the bat Doppel protein. Finally, several *in silico* tools were used to predict the impact of non-synonymous SNPs in the bat *PRND* gene.

## 2 Materials and methods

### 2.1 Ethical statements

The College of Veterinary Medicine at Jeonbuk National University donated liver and gut tissue samples from bats; these samples had been stored in a deep freezer at −80 °C before the experiment.

### 2.2 Genomic DNA

Genomic DNA was isolated from 20 mg of 98 bat tissue samples, including 11 bat species (greater horseshoe bat (*Rhinolophus ferrumequinum, n* = 58), common bent-wing bat (*Miniopterus schreibersii, n* = 13), Japanese pipistrelle (*Pipistrellus abramus, n* = 9), steppe whiskered bat (*Myotis aurascens, n* = 4), Alashanian pipistrelle (*Hypsugo alaschanicus, n* = 3), eastern water bat (*Myotis petax, n* = 3), greater tube-nosed bat (*Murina leucogaster, n* = 2), common serotine bat (*Eptesicus serotinus, n* = 2), large-footed myotis (*Myotis adversus, n* = 2), Asian parti-colored bat (*Vespertilio sinensis, n* = 1), and big-footed myotis (*Myotis macrodactylus, n* = 1)) using a Bead Genomic DNA Prep Kit (BioFACT, Daejeon, Republic of Korea). The quality and concentration of genomic DNA (15–30 ng/μl, A260/280 = 1.8–2.0) were evaluated using a NanoDrop One spectrophotometer (Thermo Scientific, Waltham, MA, USA). An A260/280 ratio of 1.8–2.0 was used as the quality criterion for DNA samples prior to sequencing.

### 2.3 Genetic analysis of the bat *PRND* gene

To amplify the bat *PRND* gene, a polymerase chain reaction (PCR) was performed with primers, including *PRND*-Forward (ACTGGAGAACTTAACCGCCC) and *PRND*-Reverse (AAAACTCGCTGGTGGTTTGC). Two primers were designed based on the greater horseshoe bat *PRND* gene (Gene ID: 117016104). The PCR mixture comprised 2.5 μl of 10 × bio *Taq* DNA polymerase buffer, 0.5 μl of 10 mM dNTP mixture, 1 μl of each primer (0.4 μM final concentration), 2.5 μl of 5 × band helper (0.5 × final concentration), 0.2 μl of *Taq* DNA polymerase (BIOFACT, Daejeon, Republic of Korea), 1 μl of genomic DNA (15–30 ng per reaction), and water to reach a total volume of 25 μl. The following experimental conditions were used for PCR: 95 °C for 2 min; followed by 32 cycles of 95 °C for 20 sec, 59 °C for 40 sec, and 72 °C for 90 sec, and 1 cycle of 72 °C for 5 min for the last extension. The PCR products were purified using the FavorPrep GEL/PCR purification kit (Favorgen Biotech, Ping Tung, Taiwan).

### 2.4 Multiple sequence alignments and evolutionary analysis

The amino acid sequences of Doppel protein were obtained from GenBank at the National Center for Biotechnology Information (NCBI), including those of humans (*Homo sapiens*, NP_036541.2), cattle (*Bos taurus*, NP_776583.1), sheep (*Ovis aries*, NP_001009261.1), goats (*Capra hircus*, XP_005688215.2), cats (*Felis catus*, XP_006929990.1), horses (*Equus caballus*, XP_005604468.1), dogs (*Canis lupus familiaris*, XP_038289147.1), rabbits (*Oryctolagus cuniculus*, XP_008254493.2), and greater horseshoe bats (*Rhinolophus ferrumequinum*, in this study). The amino acid sequences of Doppel protein were aligned using ClustalW with progressive alignment techniques. For evolutionary analysis, a phylogenetic tree according to the amino acid sequences of the Doppel protein from 9 species was constructed using MEGA version 12.1 with the maximum likelihood method (19).

### 2.5 Genetic variations of the *PRND* gene across different species

Data on polymorphisms in the *PRND* gene were gathered from previously reported studies. Genetic variations in humans, cattle, sheep, goats, cats, horses, dogs, rabbits, and bats were summarized.

### 2.6 Comparative analysis of the genomic locations of *PRNP* and *PRND* across several species

We obtained sequence information from GenBank at the NCBI. The physical map between the two genes (*PRNP* and *PRND*) was constructed using the following data. Prion diseases have been reported in humans (*Homo sapiens*, [*PRNP* Gene ID: 5621], [*PRND* Gene ID: 23627]), cattle (*Bos taurus*, [*PRNP* Gene ID: 281427], [*PRND* Gene ID: 281426]), sheep (*Ovis aries*, [*PRNP* Gene ID: 493887], [*PRND* Gene ID: 443194]), and goats (*Capra hircus*, [*PRNP* Gene ID: 102169975], [*PRND* Gene ID: 102170246]). In contrast, horses (*Equus caballus*, [*PRNP* Gene ID: 100065904], [*PRND* Gene ID: 100048937]), dogs (*Canis lupus familiaris*, [*PRNP* Gene ID: 485783], [*PRND* Gene ID: 485782]), rabbits (*Oryctolagus cuniculus*, [*PRNP* Gene ID: 100008658], [*PRND* Gene ID: 100347890]), and greater horseshoe bat (*Rhinolophus ferrumequinum*, [*PRNP* Gene ID: 117015917], [*PRND* Gene ID: 117016104]) have no reported naturally occurring cases of prion disease.

### 2.7 *In silico* models

To assess the impact of non-synonymous polymorphisms on bat Doppel protein, we used several *in silico* prediction tools, including SIFT, MutPred2, MUpro, and SODA. First, the SIFT program evaluates whether an amino acid substitution affects protein function using sequence homology and the physical characteristics of amino acids (20). The MutPred2 program uses probabilistic modeling to predict variant effects on protein structure and function, thereby aiding experimental studies of phenotype-altering variants (21). The MUpro program predicts how non-synonymous SNPs affect protein stability (22). The SODA program estimates protein solubility changes from physical and chemical properties (23).

### 2.8 AlphaFold3 for 3D structure prediction

The 3D structure of Doppel protein was predicted using AlphaFold3 based on machine learning (https://alphafoldserver.com/). The model provides confidence metrics, including the predicted local distance difference test (pLDDT), predicted aligned error (PAE), and predicted distance error (PDE). The results of the hydrogen bond alterations from the amino acid substitutions were predicted by Swiss-PdbViewer (24).

### 2.9 Statistical analyses

The minor allele frequency (MAF), LD, and haplotype results were analyzed using Haploview version 4.2 (Broad Institute, Cambridge, MA, USA). Five *PRND* polymorphisms (c.214G>A, c.391A>C, c.456G>A, c.465A>G, and c.478A>G) exhibited MAFs below 5% and were therefore classified as rare variants and excluded from LD and haplotype analyses. LD and haplotype analyses were performed using only greater horseshoe bats (*Rhinolophus ferrumequinum, n* = 58), which represented the species with the largest sample size. Hardy-Weinberg equilibrium (HWE) was evaluated using the chi-square test. A *P*-value < 0.05 was considered indicative of a significant deviation from HWE.

## 3 Results

### 3.1 Identification of 7 novel polymorphisms of the bat *PRND* gene

To identify the genetic polymorphisms of the bat *PRND* gene, we performed DNA sequencing in 98 bats. We found a total of 7 novel SNPs, including c.214G>A, c.216C>T, c.391A>C, c.392G>C, c.456G>A, c.465A>G, and c.478A>G (Figure 1; Table 1). Among them, c.216C>T (A72A), c.391A>C (R131R), c.456G>A (R152R), and c.465A>G (G155G) were synonymous SNPs, while c.214G>A (A72T), c.392G>C (R131T), and c.478A>G (M160V) were non-synonymous SNPs. These non-synonymous SNPs may potentially influence the function and structure of the Doppel protein. In Table 2, we investigated the extent of LD among the *PRND* polymorphisms using *r*^2^ values. We also investigated LD between polymorphisms located in the *PRNP* and *PRND* genes using *r*^2^ values (Table 3). No strong LD was observed among the analyzed *PRNP* and *PRND* variants. Furthermore, we performed haplotype analysis on the SNPs of the bat *PRND* gene (Table 4). The most frequent haplotype was CG (83.6%), followed by CC (9.5%) and TG (6.9%).

**Table 1:** Genotype and allele frequencies of the *prion-like* protein gene (*PRND*) polymorphisms in bats.

Polymorphisms	Genotype frequencies, *n* (%)			Allele frequencies, *n* (%)		HWE
	M/M	M/m	m/m	*M*	*m*	
c.214G>A (A72T)	97 (98.98)	0 (0)	1 (1.02)	194 (98.98)	2 (1.02)	< 0.0001
c.216C>T (A72A)	87 (88.78)	11 (11.22)	0 (0)	185 (94.39)	11 (5.61)	0.5561
c.391A>C (R131R)	89 (90.82)	9 (9.18)	0 (0)	187 (95.40)	9 (4.60)	0.6337
c.392G>C (R131T)	81 (82.65)	16 (16.33)	1 (1.02)	178 (90.82)	18 (9.18)	0.8335
c.456G>A (R152R)	94 (95.92)	4 (4.08)	0 (0)	192 (97.96)	4 (2.04)	0.8366
c.465A>G (G155G)	97 (98.98)	1 (1.02)	0 (0)	195 (99.49)	1 (0.51)	0.9595
c.478A>G (M160V)	97 (98.98)	1 (1.02)	0 (0)	195 (99.49)	1 (0.51)	0.9595

M/M, major homozygote; M/m, heterozygote; m/m, minor homozygote; M, major allele; m, minor allele; HWE, Hardy–Weinberg equilibrium.

**Table 2:** Linkage disequilibrium (LD) analysis of the *PRND* polymorphisms in bats.

Polymorphisms	c.216C>T	c.392G>C
c.216C>T	–	–
c.392G>C	0.006	–

The *PRND* polymorphisms with a minor allele frequency (MAF) below 5% were excluded from the LD analysis.

**Table 3:** Linkage disequilibrium (LD) analysis between polymorphisms of the prion protein gene (*PRNP*) and *prion-like* protein gene (*PRND*) in bats.

Polymorphisms	*PRND* c.216C>T	*PRND* c.392G>C
*PRNP* c.86A>G	0.001	0.001
*PRNP* c.156C>T	0.001	0.001
*PRNP* c.216T>C	0.006	0.008
*PRNP* c.219T>C	0.042	0.016
*PRNP* c.300A>G	0.012	0.021

The vertical and horizontal axes represent *PRNP* and *PRND* polymorphisms, respectively.

**Table 4:** Haplotype frequency of common *PRND* polymorphisms in bats.

Haplotype	Frequency, *n* (%)
CG	97 (83.6)
CC	11 (9.5)
TG	8 (6.9)
Total	116 (100)

**Figure 1:**
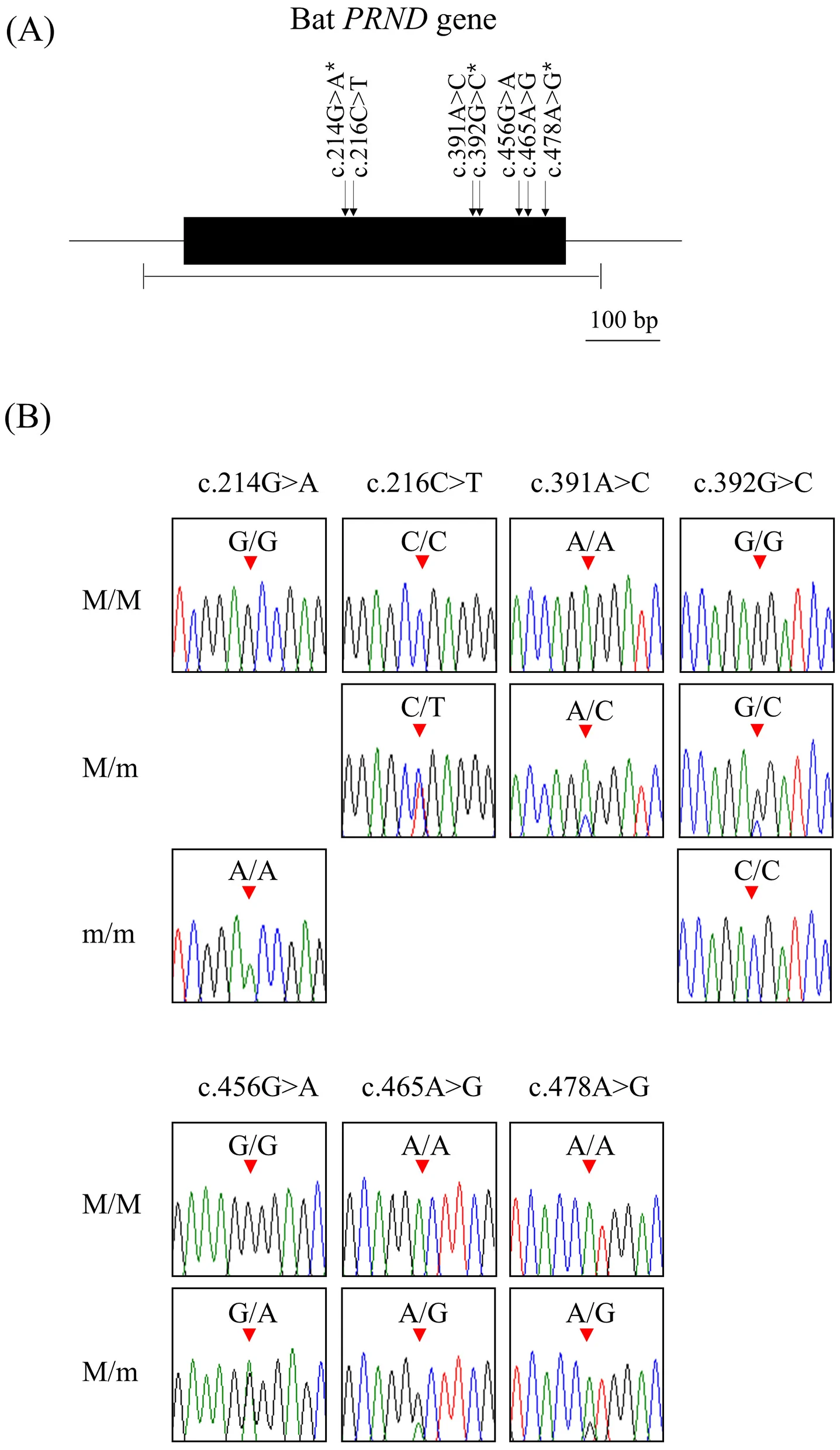
Identification of single nucleotide polymorphisms (SNPs) in the bat *PRND* gene. **(A)** The gene diagram displays the genomic structure of the *prion-like* protein gene (*PRND*). The open reading frame (ORF) is depicted as a black box. Asterisks represent non-synonymous SNPs. **(B)** Electropherograms display 7 novel SNPs identified in the bat *PRND* gene. The symbol M/M represents major homozygotes, M/m represents heterozygotes, and m/m represents minor homozygotes.

### 3.2 Multiple sequence alignment and evolutionary analysis of the amino acid sequences of Doppel protein

We performed multiple sequence alignments of amino acid sequences of Doppel protein among humans, cattle, sheep, goats, cats, horses, dogs, rabbits, and bats (Figure 2A). When compared to other species, the bat Doppel protein contained six specific amino acids, including glutamine (Q) at codon 4, arginine (R) at codons 25 and 51, methionine (M) at codon 54, serine (S) at codon 163, and valine (V) at codon 175.

**Figure 2:**
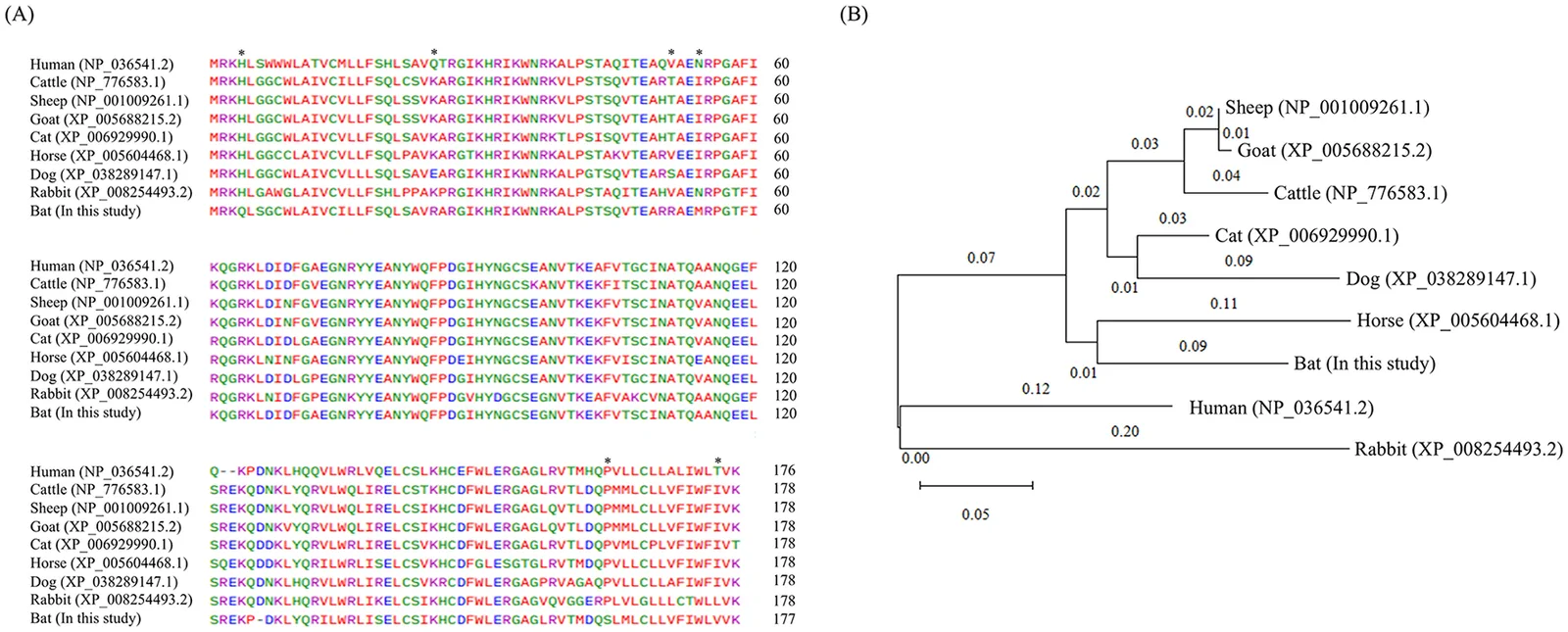
Multiple sequence alignment and evolutionary analysis of the amino acid sequences of Doppel protein. **(A)** Multiple sequence alignment of Doppel in various animals. Several colors symbolize the chemical properties of amino acids (red: tiny and hydrophobic; blue: acidic; violet: basic; green: hydroxyl, sulfhydryl, amine and glycine). Asterisks represent bat-specific amino acids. **(B)** Phylogenetic tree of several species for evolutionary analysis.

Next, to evaluate the phylogenetic relationships between bats and prion disease-susceptible species, we constructed a phylogenetic tree (Figure 2B). Notably, the branch length between bats and horses (XP_005604468.1) was approximately 0.09–0.11. The bats identified formed a unique cluster with horses. Although this clustering indicates phylogenetic relatedness between the Doppel sequences of bats and horses, it does not provide evidence of a shared prion-resistance phenotype.

### 3.3 Comparison of the *PRND* polymorphisms across various species

To compare the distribution of genetic polymorphisms, we analyzed the open reading frame (ORF) of the *PRND* gene across several species (Figure 3). Similar Doppel amino acid sequence lengths were found in bats and other species (around 176–178 amino acids). There were no insertion/deletion polymorphisms across all species examined.

**Figure 3:**
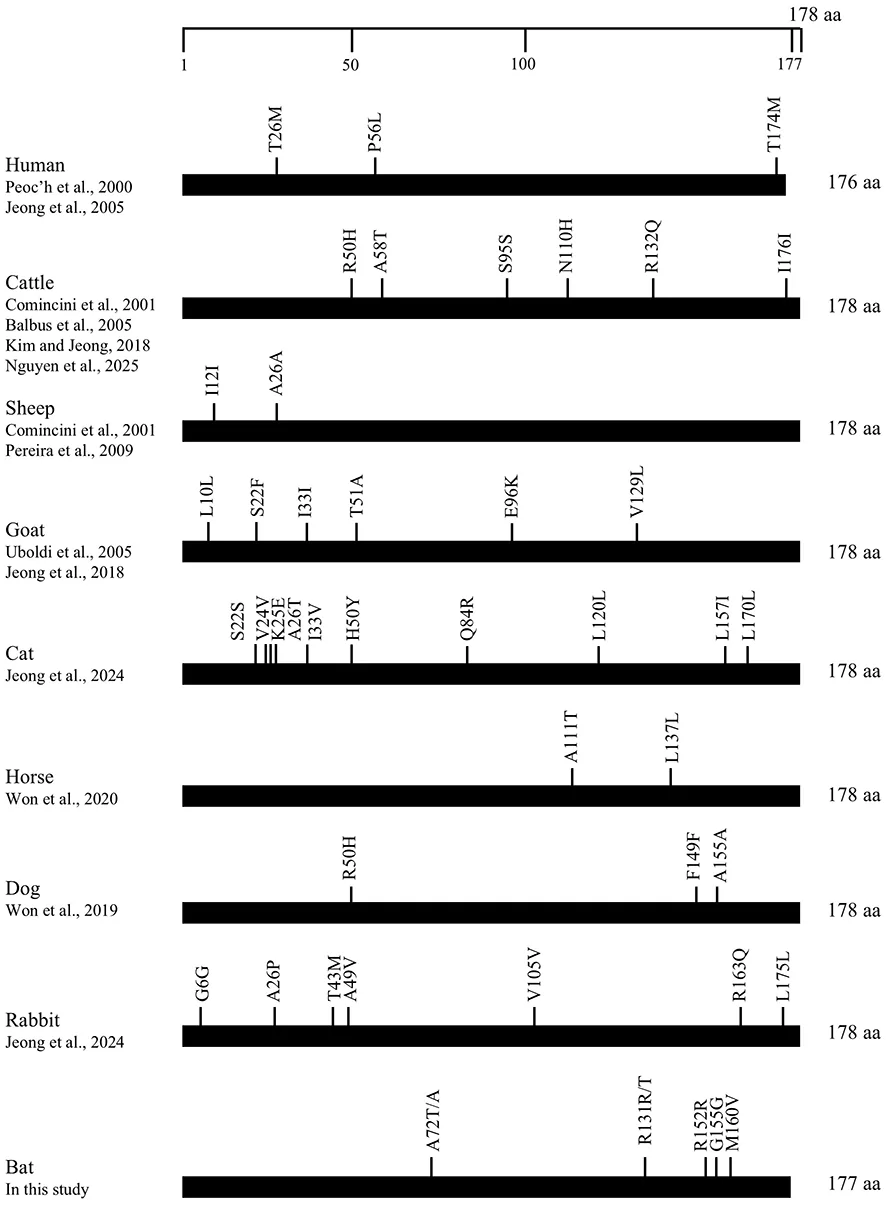
Distribution of genetic polymorphisms in the open reading frame (ORF) of the *PRND* gene across various species. The figure illustrates the identified genetic polymorphisms of the *PRND* gene in various species, including humans (45, 46), cattle (8, 12, 47, 48), sheep (8, 49), goats (27, 50), cats (11), horses (10), dogs (28), rabbits (51), and bats. The solid horizontal bar represents the length of the Doppel amino acid sequence.

### 3.4 Measurement of the physical distance between the two genes (*PRNP* and *PRND*) among several species

The physical distance between two genes (*PRNP* and *PRND*) was measured in several species such as humans, cattle, sheep, goats, horses, dogs, rabbits, and bats (Supplementary Figure 1). Notably, prion disease-resistant species, such as dogs (17 kb), horses (16 kb), and rabbits (12 kb), showed relatively short physical distance between two genes. In contrast, prion disease-susceptible animals, including humans (20 kb), cattle (25 kb), sheep (25 kb), and goats (25 kb), generally exhibited longer distances. In greater horseshoe bats (*Rhinolophus ferrumequinum*), the physical distance between the two genes was short, measuring 14 kb.

### 3.5 *In silico* analysis of the effect of non-synonymous SNPs in bats

To measure the potential effects of three non-synonymous SNPs, we used SIFT, MutPred2, MUpro, and SODA (Table 5). The SIFT results indicated that c.214G>A (A72T) and c.478A>G (M160V) were predicted to be tolerated, while c.392G>C (R131T) was predicted to be damaging. Similarly, the MutPred2 results classified c.214G>A (A72T) and c.478A>G (M160V) as benign, while c.392G>C (R131T) was predicted to be pathogenic. The MUpro and SODA analyses indicated that these three non-synonymous SNPs are expected to decrease protein stability and solubility, respectively.

**Table 5:** *In silico* evaluation of the potential effects of non-synonymous SNPs in bats.

Polymorphisms	Method	Score	Prediction
c.214G>A (A72T)	SIFT	0.200	Tolerated
	MutPred2	0.472	Benign
	MUpro	−0.937	Decrease
	SODA	−0.096	Less soluble
c.392G>C (R131T)	SIFT	0.040	Damaging
	MutPred2	0.526	Pathogenic
	MUpro	−1.497	Decrease
	SODA	−21.973	Less soluble
c.478A>G (M160V)	SIFT	0.510	Tolerated
	MutPred2	0.152	Benign
	MUpro	−1.398	Decrease
	SODA	−82.835	Less soluble

### 3.6 Prediction of the 3D structures of bat Doppel protein

We predicted the 3D structures of the wild-type and non-synonymous variant Doppel proteins and analyzed their hydrogen-bond interactions. The A72 and T72 residues were predicted not to form hydrogen bonds with surrounding residues (Figure 4A). The R131 and T131 residues were each predicted to form three hydrogen bonds with K127 (3.08 Å), L128 (3.29 Å), and R135 (3.10 Å) (Figure 4B). The M160 and V160 residues were each predicted to form one hydrogen bond with L164 (3.08 Å) (Figure 4C). No marked differences in the local hydrogen-bonding patterns were observed between the wild-type and variant residues.

**Figure 4:**
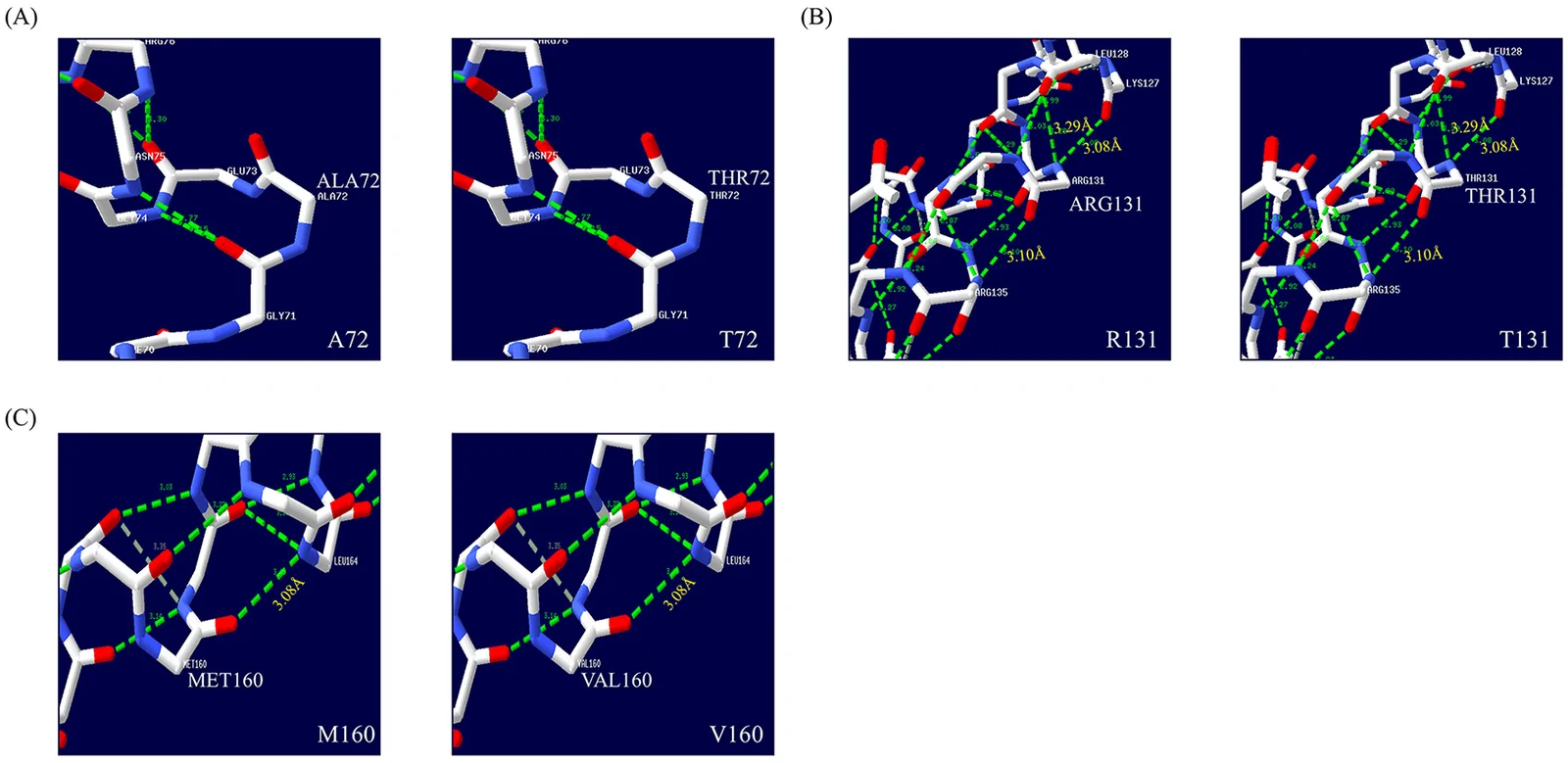
The 3D structures of the Doppel protein with non-synonymous SNPs. **(A)** SNP c.214G > A (A72T). **(B)** SNP c.392G > C (R131T). **(C)** SNP c.478A > G (M160V).

## 4 Discussion

Considering that the pathological pathways of prion diseases remain largely unknown, investigating the genetic characteristics of species either susceptible or resistant to these diseases is crucial for an in-depth understanding. We extended our previous study with the same bat samples, which reported polymorphisms in the bat *PRNP* and *SPRN* genes (25, 26). The *PRNP* results provided preliminary genetic insights potentially relevant to natural resistance to prion diseases in bats. In this study, we focused on identifying polymorphisms within the bat *PRND* gene. We found a total of seven SNPs within its ORF (Figure 1; Table 1). However, due to the relatively small and unequal sample sizes among bat species, the statistical power of allele frequency calculations and population genetic estimates, including HWE tests, may be limited. In particular, c.214G>A showed a marked deviation from HWE (*P* < 0.0001). This deviation may be attributable, at least in part, to the heterogeneous composition of the sampled bats, which included multiple species with unequal sample sizes, resulting in population stratification. In addition, the very low minor allele frequency of c.214G>A may reduce the reliability of the HWE estimate. Although sequencing artifacts cannot be completely excluded, this deviation should be interpreted cautiously and should not be regarded as direct evidence of biological selection. Therefore, these genetic analyses should be interpreted with caution. Furthermore, because the present study focused only on the ORF, future studies should also examine the 3′ UTRs and promoter regions.

Phylogenetic analysis showed that bat Doppel sequences clustered most closely with those of horses (Figure 2B). Although horses have been regarded as a prion-resistant species, this phylogenetic relationship does not indicate a shared resistance phenotype. Furthermore, we investigated the genetic linkage between variants located in the bat *PRNP* and *PRND* genes and observed weak LD among the analyzed variants (Table 3). This is notable because prion disease-susceptible species (goats) show strong LD between polymorphisms of these two genes (27), whereas prion disease-resistant species (dogs) exhibit weak LD (28). The weak LD between *PRNP* and *PRND* variants reflects limited allelic association within the analyzed population and should not be interpreted as evidence of independent regulation of these genes. Additionally, insertion/deletion polymorphisms have been reported in the upstream and downstream regions of the *PRND* gene in prion disease-susceptible species (sheep and cats) (11, 29). Consistent with observations in bats, no insertion/deletion polymorphisms were detected within the ORF across any of the other species examined (Figure 3). We also summarized the physical map distance between the two genes. The *PRND* gene is located downstream of the *PRNP* gene (4, 30). The greater horseshoe bat *PRND* gene is located downstream of the bat *PRNP* gene at an intergenic distance of approximately 14 kb (Supplementary Figure 1). Although a relatively short physical distance was observed, this may reflect genomic organization rather than a direct functional association. Taken together, these genetic observations provide a basis for further investigation of potential prion resistance in bats, but they do not constitute direct evidence of a resistant phenotype. Experimental validation, including molecular dynamics simulations and *in vitro* functional assays, is therefore required.

Next, the non-synonymous SNP c.392G>C (R131T) was predicted to be deleterious to Doppel protein function by two tools (Table 5). However, no marked structural differences or changes in local hydrogen-bonding patterns were observed among the AlphaFold3-predicted models associated with non-synonymous SNPs (Figure 4). A previous study reported, using molecular dynamics simulation, that mutations can perturb hydrogen-bond interactions involving residues within the native β-sheet in human PrP (31). Additionally, *in silico* analysis of protein structure and function has been widely conducted across various genes, including those implicated in neurodegenerative disorders (32–35). While SNP-based predictions offer valuable insights, current *in silico* analyses have limitations in fully capturing functional and structural changes. As predictive modeling advances, accurately defining these functional consequences is becoming increasingly important for broader biosafety assessments (36). Furthermore, functional validation (*in vitro* or *in vivo*) of the identified SNPs was not performed in this study, which limits the definitive interpretation of their biological significance. Therefore, empirical experimental studies are required to investigate how various SNPs influence Doppel's structure and function, ultimately elucidating their roles in prion disease pathophysiology and broader biological contexts.

Regarding horses, no polymorphisms have been found in Thoroughbred horses, while two polymorphisms have been reported in the *PRND* gene of Korean native horses (9, 10). The genetic distribution of the gene may be biased depending on the mating environment. Bats are uniquely interesting to researchers because they exhibit a greater diversity in mating systems than any other mammal (37). Doppel protein was previously known to be predominantly expressed in the male reproductive organs (5, 38), but recent studies have highlighted its role as a biomarker for ovarian cancer (39). Additionally, Doppel protein helps recovery after ischemic stroke (40). It is necessary to determine whether this complex mating system can also influence gene expression, particularly the expression patterns of proteins associated with reproduction and other diseases.

Bats comprise numerous species, with over 1,400 known (41). While a recent study has suggested investigating the potential role of bats in the transmission of chronic wasting disease to sympatric mammals (42), alongside their established status as viral vectors (43), the implication that bats pose a direct risk for prion transmission must be substantially qualified. Although no prion infection has been reported in bats to date, the present study does not provide evidence that bats pose a direct risk for prion acquisition, replication, or transmission. Future studies should include functional assays and broader species comparisons to validate the contribution of *PRND* polymorphisms in prion disease resistance. Moreover, a recent investigation into prion-like sequence features in coronaviruses illustrates the broader application of computational approaches for characterizing prion-like domains and sequence-dependent structural tendencies in infectious agents (44). Although such viral prion-like sequence features are biologically distinct from the mammalian *PRND*/Doppel system investigated here, these studies highlight the broader utility of computational analyses for examining conformationally relevant sequence features in diverse biological contexts.

## 5 Conclusion

In conclusion, we found a total of 7 novel SNPs in bats, including three non-synonymous SNPs: c.214G>A (A72T), c.392G>C (R131T), and c.478A>G (M160V). We also evaluated the LD between SNPs located in the two genes (*PRNP* and *PRND*) and detected weak LD among the analyzed variants. Finally, the one non-synonymous SNP c.392G>C (R131T) was predicted to have a potentially deleterious effect on the Doppel protein based on preliminary *in silico* analyses. Future molecular dynamics simulations and *in vitro* assays are needed to determine how these novel variants affect protein stability, surface expression, and neurotoxicity.

## Data Availability

All data are available from the corresponding author upon reasonable request. All sequence meta-data generated in this study have been deposited in the National Center for Biotechnology Information (NCBI) GenBank repository under accession number PZ268213.1.
